# Transcriptome analysis of human dermal fibroblasts following red light phototherapy

**DOI:** 10.1038/s41598-021-86623-2

**Published:** 2021-04-01

**Authors:** Evan Austin, Eugene Koo, Alexander Merleev, Denis Torre, Alina Marusina, Guillaume Luxardi, Andrew Mamalis, Roslyn Rivkah Isseroff, Avi Ma’ayan, Emanual Maverakis, Jared Jagdeo

**Affiliations:** 1 Department of Dermatology, University of California at Davis, Sacramento, CA USA; 2 Department of Dermatology, SUNY Downstate, Brooklyn, NY USA; 3 Department of Pharmacological Sciences, Mount Sinai Center for Bioinformatics, Icahn School of Medicine at Mount Sinai Health, New York, NY USA; 4 Dermatology Service, Sacramento VA Medical Center, Mather, CA USA

**Keywords:** Transcriptomics, Skin diseases

## Abstract

Fibrosis occurs when collagen deposition and fibroblast proliferation replace healthy tissue. Red light (RL) may improve skin fibrosis via photobiomodulation, the process by which photosensitive chromophores in cells absorb visible or near-infrared light and undergo photophysical reactions. Our previous research demonstrated that high fluence RL reduces fibroblast proliferation, collagen deposition, and migration. Despite the identification of several cellular mechanisms underpinning RL phototherapy, little is known about the transcriptional changes that lead to anti-fibrotic cellular responses. Herein, RNA sequencing was performed on human dermal fibroblasts treated with RL phototherapy. Pathway enrichment and transcription factor analysis revealed regulation of extracellular matrices, proliferation, and cellular responses to oxygen-containing compounds following RL phototherapy. Specifically, RL phototherapy increased the expression of *MMP1*, which codes for matrix metalloproteinase-1 (MMP-1) and is responsible for remodeling extracellular collagen. Differential regulation of *MMP1* was confirmed with RT-qPCR and ELISA. Additionally, RL upregulated *PRSS35,* which has not been previously associated with skin activity, but has known anti-fibrotic functions. Our results suggest that RL may benefit patients by altering fibrotic gene expression.

## Introduction

As part of the healing process, fibroblasts proliferate, differentiate, and increase collagen production^1,2^. Fibrosis occurs when collagen deposition and fibroblast proliferation replace healthy tissue due to impaired wound healing, immune dysfunction, or iatrogenic causes^3^. Fibrosis can affect most organs, including the skin, liver, ovaries, and lungs^3–7^. Keloids, hypertrophic scars, scleroderma, and radiation dermatitis are among the many manifestations of skin fibrosis that significantly burden patients' quality of life^8,9^. Current treatment options include 5-fluorouracil, radiation therapy, immunomodulators, and surgery, but fibrosis is often recalcitrant to existing approaches^10^. Ultraviolet-A1 (UVA1; 340–400 nm) phototherapy is an existing treatment for fibrosis but has a limited depth of penetration (less than 150 μm) and carries a risk for non-melanoma skin cancer and aging^11–14^. Red light (RL) may be a potentially safer alternative to UVA1 with a greater depth of penetration (6–50 mm)^15–18^. RL may improve skin fibrosis via photobiomodulation, the process by which photosensitive chromophores in skin cells absorb visible or near-infrared light and undergo photophysical reactions^19,20^. Photobiomodulation was previously known as low-level light therapy, but higher fluences (i.e., a higher amount of light energy delivered over the treatment period) may also induce cellular changes.

Photobiomodulation has a multiphasic response in which lower fluences are stimulatory, while higher fluences are inhibitory or cytotoxic^21^. Much of the existing research has focused on the effects of visible or near-infrared light at fluences lower than 150 J/cm^2^ to stimulate cell growth for skin rejuvenation or hair growth^22^. However, our laboratory team has studied the inhibitory properties of red light greater or equal to 320 J/cm^2^ to treat pathological hyperproliferative processes, including fibrosis^23–25^. Our previous research demonstrated that photobiomodulation using high fluence RL reduces human dermal fibroblast (HDF) proliferation, collagen deposition, and migration in vitro^23–25^. We sought to investigate changes in gene expression following RL phototherapy. We performed high-throughput RNA sequencing (RNA-Seq) to identify genes and pathways associated with RL. RNA-Seq allows for unbiased discovery of therapeutic targets and whole transcriptome gene expression analysis.

## Results

### Transcriptomic profiling of human dermal fibroblasts

**Figure 1:**
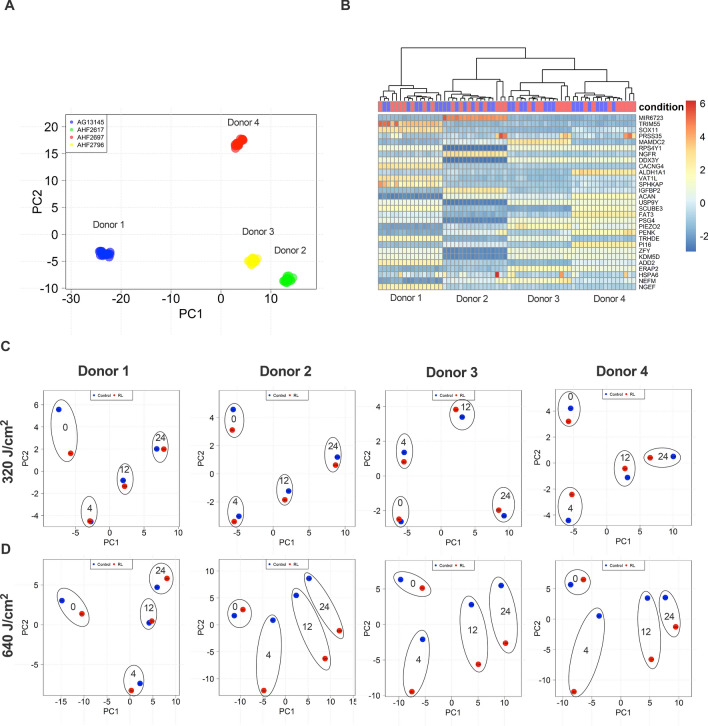
Characteristics of HDF Donors following RL treatment. RL treated and control samples cluster according to donor and time. **(A)** Combined sample donor PCA demonstrating clustering within donors. PCA plots are regularized log-transformed data and were created with the DESeq2 bioconductor R package. The original count data was transformed to the log_2_ scale to minimize differences between samples for rows with small counts, normalizing according to library size. After transformation, the top 500 rows with the highest variance were used for further principal component analysis. **(B)** Heat map cluster of a subset of the top 30 most highly variable genes. Blue and red bars on the condition row represent control and RL treated samples, respectively. **(C)** PCA of individual sample donors 1–4 for 320 J/cm^2^ and (D) 640 J/cm^2^ fluences demonstrates clustering of 0-h time points from 4, 12, and 24 h time points. Control (blue) and RL (red) treated samples are circled and labeled by time point (i.e., 0, 4, 12, and 24 h).

Principal component analysis (PCA) demonstrated that samples segregate according to donors, which is characteristic of human subjects’ analysis (Table 1 and Fig. 1A). The heat map of the top 30 genes with maximum variance values, calculated for all samples, shows similar segregation by donor line (Fig. 1B). When PCA analysis was separately performed for each donor, samples collected at the 0-h timepoint clustered apart from the 4, 12, and 24 h (Fig. 1C,D). 640 J/cm^2^ treated samples clustered separately from control for 3 out of 4 donor lines (Fig. 1D). It should be noted that changes between treatment and control were found to be in the same direction of gene expression dimensionality reduced space.

**Table 1:** HDF donor characteristics.

Designation	Donor 1	Donor 2	Donor 3	Donor 4
Name	AG13145	CRL-2617	CRL-2697	CRL-2796
Vendor	Coriell	ATCC	ATCC	ATCC
Anatomical Site	Forearm	Abdomen	Leg	Abdomen
Age	57	42	36	44
Gender	Male	Female	Male	Male
Ethnicity	Caucasian	African–American	Caucasian	Caucasian

To account for interpersonal variations in gene expression, DESeq2, capable of paired analysis, was used to identify differentially expressed genes (DEGs)^26^. Analysis of the samples revealed 859 DEGs following RL with a twofold change in expression and false discovery rate (FDR) < 0.05 across all timepoints. The complete list of DEGs from the analysis with fold change and FDR can be found in the supplemental dataset.

### RL induces a temporal change in gene expression

**Figure 2:**
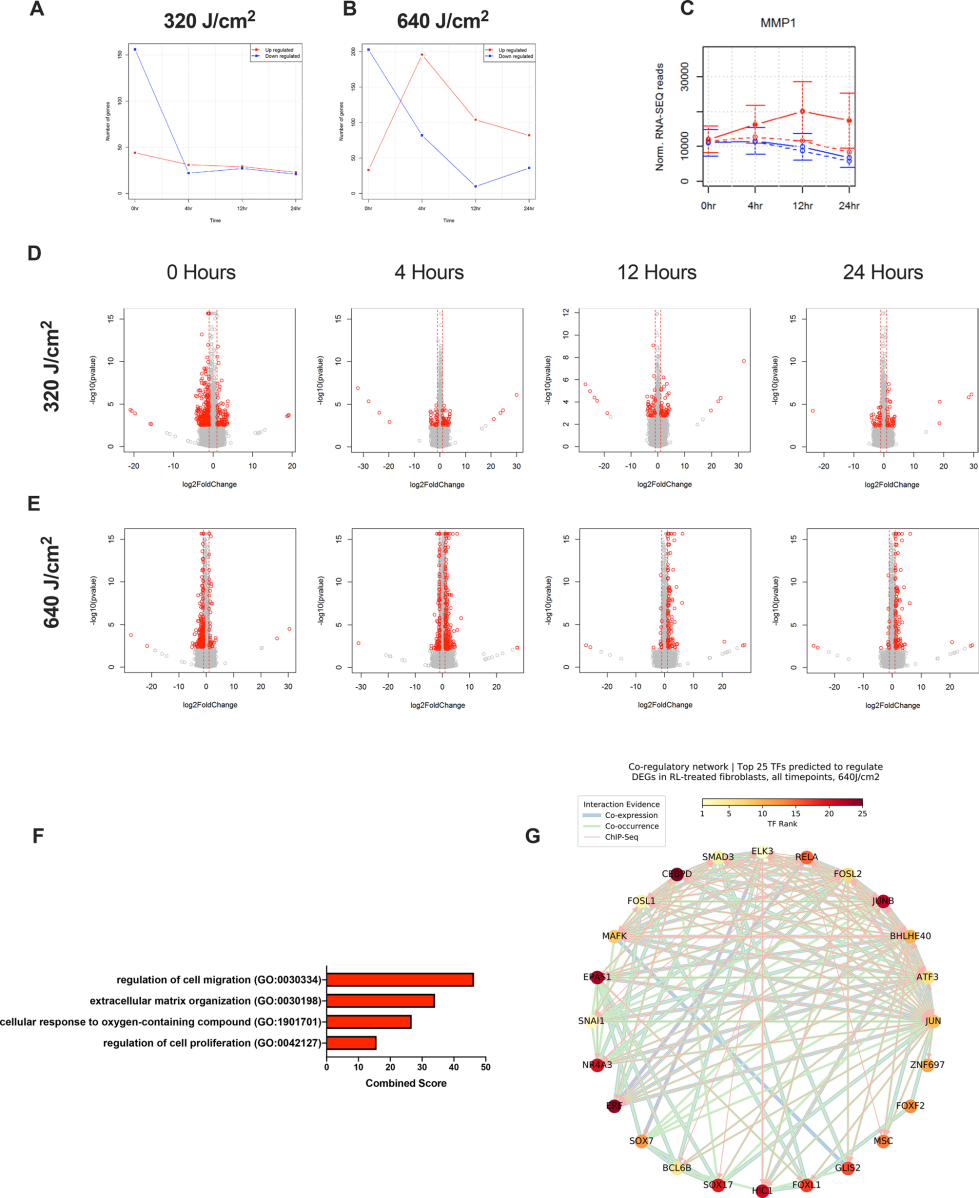
Transcriptomic and pathway enrichment profile of HDFs. The most considerable change in gene expression occurs between 0 and 4 h. **(A)** The number of up (red) and down (blue) DEGs by time for the 320 J/cm^2^ and **(B)** 640 J/cm^2^ fluences. **(C)** Representative expression profile of *MMP1.* Dashed and solid lines represent the 320 and 640 J/cm^2^ fluences, respectively. Blue lines are control samples, and red lines are RL treated samples. **(D)** Volcano plots of DEGs following irradiation with 320 J/cm^2^ and**(E)** 640 J/cm^2^ fluences. Red dots are DEGs with FDR-adjusted p values < 0.05 and fold change > 2 or < 0.5. Grey dots have FDR-adjusted p values > 0.05 or fold change between 2 and 0.5. DESeq2 was used to calculate DEGs for 320 J/cm^2^ and 640 J/cm^2^ RL treated samples. The number of DEGs was calculated by averaging the results of all four donor samples. Differential expression analysis was performed for each time point and the p-values were corrected for multiple testing using the FDR method. **(F)** Enriched GO pathways of interest, in 640 J/cm^2^ RL, all timepoints. **(G)** Co-regulatory networks of top 25 TF predicted to regulate differential expression in 640 J/cm^2^ RL treated HDFs and pooled time points.

At 0 h after RL, 147 out of 191 and 205 out of 239 DEGs were downregulated for both 320 J/cm^2^ and 640 J/cm^2^, respectively (Figs. 2A,B). Four hours post-treatment, the trend reversed for HDFs treated with 640 J/cm^2^ RL as 304 DEGs were upregulated (Fig. 2B). Four hours was the temporal apex of DEG upregulation (Fig. 2B). 640 J/cm^2^ RL may cause immediate downregulation of transcription and subsequent upregulatory cellular compensations. At 320 J/cm^2^, the number of downregulated DEGs decreased such that up and downregulation were approximately equal at 4 (28 up/17 down), 12 (19 up/18 down), and 24 h post-treatment (22 up/19 down) (Fig. 2A). These results indicate that the greatest changes in gene expression occur within 4 h and is dose-dependent. The differential expression of *MMP1* (fold change: 2.36, p-value: 9.86 × 10^–14^, FDR: 5.02 × 10^–11^ at 24 h) is shown as a representative gene of interest involved in extracellular matrix organization (Fig. 2C). Volcano plots of fold-changes versus significance demonstrate that some highly expressed genes did not have an FDR greater than 0.05, likely due to a small sample size of fibroblast donors (Fig. 2D,E). 640 J/cm^2^ induced significant enrichment of the upregulation of cell migration (GO: 0030334, p-value: 4.39 × 10^–7^, q-value: 2.24 × 10^–3^), extracellular matrix organization (GO: 0030198, p-value: 1.96 × 10^–5^, q-value: 9.11 × 10^–3^), cellular response to oxygen-containing compounds (GO:1901701, p-value: 6.53 × 10^–5^, q-value: 1.67 × 10^–2^), and regulation of cell proliferation (GO: 0042127, p-value: 2.49 × 10^–4^, q-value: 4.23 × 10^–2^) by GO analysis via Fisher exact test (Fig. 2F). Complete GO and KEGG pathway analyses are provided in Figures S1-4. Transcription factor (TF) associated with oxidative stress (e.g., *RELA*) and fibrosis (*SMAD3, FOSL1, FOSL2, JUN, and JUNB*) are predicted to regulate the cellular response to RL phototherapy (Fig. 2G)^27–31^.

### Identification of genes associated with fibrosis and oxidative stress

We aimed to investigate transcriptional changes induced by RL in HDFs. To determine if RL photobiomodulation induces differential expression beneficial for the treatment of fibrotic skin disease, candidate genes associated with fibrosis were further analyzed. The following DEGs were identified as related to fibrotic processes: *DOCK4, WNT2, MMP1, PSAT1, MIR21, MIR145, and BTBD11* (Figure S5). *PRSS35, RANBP3L*, and *MECOM* had higher than tenfold changes in expression and are associated with fibrosis. The data was reanalyzed without a fold-change cutoff, and we observed differential expression of *SMAD3*, *SMAD4*, and *SMAD7* (Figure S6). Additionally, we searched for candidate gene transcripts related to reactive oxygen species (ROS) regulation. ROS has been associated with photobiomodulation’s mechanism and changes in apoptotic, cytoprotective, and fibrotic pathways^22,25^. Identified ROS related DEGS were *AKR1B1, NPAS2, AKR1C1, RCAN1, MRPS6, XDH, ADM2, NCF2, DDIT4L, SLC5A3,* and *GUCY1A2* (Figure S7).

### Confirmation of differential expression and ontology

**Figure 3:**
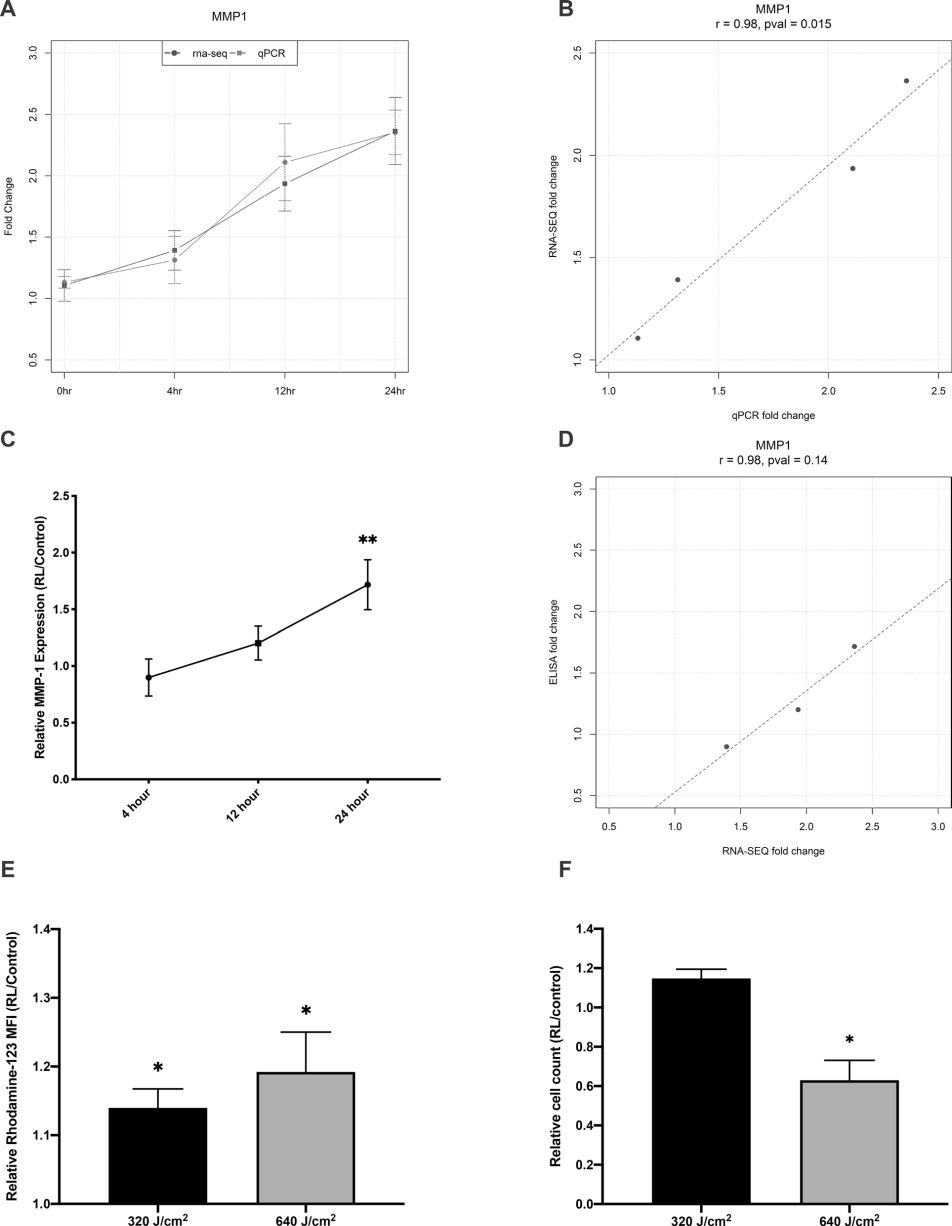
Validation of RNA-seq results. **(A)** RT-qPCR was performed with samples from the donors 1–4. RNA for RT-qPCR and RNA-Seq was collected separately. Red and blue bars represent fold-change for *MMP1* from RT-qPCR and RNA-Seq, respectively **(B)** Pearson correlation of *MMP1* differential expression between RT-qPCR and RNA-Seq show high (R = 0.98) and significant correlation (p < .05). **(C)** MMP-1 protein secretion confirmation of RNA-Seq. Culture supernatant was collected from RL and control samples from all four donors at 4, 12, and 24 h post-irradiation. MMP-1 protein secretion was quantified using ELISA **(D)** Pearson correlation between MMP-1 and ELISA show high (R = 0.98), but not significant correlation (p > .05). **(E)** 320 and 640 J/cm^2^ RL immediately increased ROS generation as assessed by rhodamine-123 MFI. Following RL phototherapy, HDFs were stained with DHR-123 (which converts to rhodamine-123 in the presence of ROS) for 30 min. HDF were collected and MFI was measured using flow cytometry **(F)** 640 J/cm^2^ decreased cell counts as assessed by crystal violet elution. Following RL, HDFs were fixed and stained with crystal violet. The optical density of eluted crystal violet served as a proxy for cell count. For each donor, the MMP-1 ELISA, ROS flow cytometry, and cell counts experiments were performed with a technical repeat of at least 3. Relative (RL/control) MMP-1 expression, rhodamine-123 MFI, and cell counts were pooled from the 4 donor lines and compared to a hypothetical mean of 1 (indicating no difference between RL and control), using a one sample T-Test. P < .05 (*) was considered significant.

Verification of *MMP1* protein and gene expression was performed (Figs. 3A–D). *MMP1* produces matrix metalloproteinase-1 (MMP-1), also known as collagenase, and is capable of enzymatically degrading the collagen found in fibrosis^32,33^. Changes in gene expression between RT-qPCR and RNA-Seq were in the same direction and had similar fold changes at the 0, 4, 12, and 24 h time points (Figs. 3A). A Pearson correlation statistical test was performed to determine consistency between RT-qPCR and RNA-Seq found an R = 0.98 and a significant P-value of 0.015 (Fig. 3B). At 4, 12, and 24 post-RL irradiation, the supernatant was collected from the control and RL-treated sample. MMP-1 protein expression was quantified using ELISA. By 24 h post-treatment, RL treated samples released significantly more MMP-1 compared to control (Fig. 3C). A Pearson correlation statistical test to determine consistency between MMP-1 ELISA and RNA-Seq differential expression was performed. RNA-Seq and ELISA were highly (R = 0.98), but not significantly (P = 0.14), correlated (Fig. 3D).

We previously found that RL increased intracellular ROS generation and inhibited cell proliferation in HDF donor 1^25^. GO pathway analysis revealed enrichment of cellular responses to oxygen containing compounds and regulation of cell proliferation in all 4 donors treated with 640 J/cm^2^ (Fig. 2F). We confirmed that ROS increases and cell count decreases in donors 1–4 when treated with RL. 320 and 640 J/cm^2^ RL increased ROS in a dose dependent manner at 0 h post-treatment (Fig. 3E). 640 J/cm^2^ RL decreased cell count by 48 h post irradiation (Fig. 3F).

## Discussion

We present whole transcriptome gene expression analysis of HDFs at 0, 4, 12, and 24 h after treatment with 320 or 640 J/cm^2^ RL. There were more DEGs within 4 h of treatment than by 12 and 24 h after RL treatment (Fig. 2A). This is consistent with our previous finding that RL mediated effects on HDF migration dissipated within 12 h^25^. *MMP1* expression was confirmed with RT-qPCR and ELISA for *MMP1*. Additionally, *PRSS35*, which produces a serine protease with collagen 1 degrading potential, was found to have greater than 30 fold increased expression at 4, 12, and 24 h after RL treatment, and thus may impart anti-fibrotic effects via collagen degradation^34^. This study provides foundational insights for future investigation into photobiomodulation and fibrosis.

We have previously shown that RL directly decreases collagen protein expression^25^. GO pathway analysis demonstrated enrichment of genes related to the organization of the extracellular matrix (Fig. 2F). As a result, we sought to investigate genes and pathways associated with anti-fibrotic activity. Other researchers have found that skin MMP-1 expression and secretion increases in response to UV and visible light^35,36^. Li et al. found that 3 J/cm^2^ RL increased the expression of multiple MMPs, including MMP-1^37^. We confirmed similar increases in MMP-1 expression and secretion in HDF treated with high fluence RL (Fig. 3). This is significant as RL is not associated with skin cancer and aging like UVA phototherapy.

*PRSS35* is a serine protease that may have collagen-1 degrading function^34^. PRSS35 has been previously linked to gonadal function, but human epididymis protein 4 (HE4), an inhibitor of PRSS35, was highly expressed in fibrotic kidneys^34,38,39^. We observed a greater than 30-fold increase of *PRSS35* expression in the RL treated HDFs, suggesting that PRSS35 induction by RL may reduce collagen. The increased expression of collagen-1 degrading enzymes may highlight the anti-fibrotic mechanisms of RL through the degradation of extracellular collagen.

To further characterize the potential of RL in fibrosis, DEGs in the TGF-β signaling pathway were analyzed (Figure S3).^40^. We have previously shown that 640 J/cm^2^ RL decreases SMAD-2 phosphorylation in TGF-β1 induced HDFs within 4 h of irradiation^41^. Phosphorylated SMAD 2/3 translocate to the nucleus and increase the expression of collagen^42–44^. In our RNA-Seq analysis, HDFs were not TGF-β induced, but there was nevertheless downregulation of profibrotic *SMAD3* at 4, 12, and 24 h post-RL treatment. TF analysis confirmed that *SMAD3* was likely involved with regulation of cellular activity following RL phototherapy (Fig. 2G). SMAD-4 is pro-fibrotic, but its expression was increased at 4 h post-RL treatment^45^. SMAD-7 has anti-fibrotic properties and was slightly downregulated in our RNA-seq data^46,47^.

There were other highly expressed DEGs involved with SMAD regulation. *RANBP3L* produces a protein that acts as a nuclear factor that can export SMAD-1 and other proteins known to have anti-fibrotic properties as part of the TGF-β pathway^48^. *MECOM* produces a protein called *EVI-1*, which acts as a transcriptional regulator that can inhibit SMAD protein activity^49,50^.

We previously performed RNA-Seq for miRNA from HDF donor line 1 and found that miRNA-21, miRNA-23, and miRNA-31 were decreased, while miRNA-29, miRNA-196a, and let-7a were increased^51^. These microRNAs have been identified as mediators of skin fibrosis^52^. We repeated our miRNA analysis with HDF donor lines 1–4 and confirmed that miRNA-21 (*Mir21*) expression was significantly downregulated following RL irradiation (Figure S2). miRNA-21 regulates TGF-β/SMAD signaling, and decreased expression of miR-21 is anti-fibrotic^52^. Additionally, miRNA-145 expression was decreased. miRNA-145 is increased in hypertrophic scars and has been identified as a therapeutic target for anti-fibrotic therapies^53^.

FOS/JUN family of proteins (i.e., *FOSL1, FOSL2, JUNB,* and *JUN*) were identified as TF regulating the response of fibroblasts to RL phototherapy. JUN proteins form homodimers or heterodimers with FOS proteins to increase the expression of AP1^28,30^. AP1 can regulate cell cycle progression and extracellular matrix organization^28^. c-Jun (*JUN*) is phosphorylated by c-Jun N-terminal kinases (JNKs) in response to cellular stress, growth factors, or cytokines^28^. c-Jun is important in IL-17 mediated production of MMP-1 in HDFs^28,29^. Similarly, inhibition of JNK in HDFs prevented the upregulation of MMP-3 and MMP-1 in response to UVB^28,31^. Fibrotic responses to FOS/JUN activity can change based on cell type and conditions^28–30^. c-Jun is upregulated in the skin of patients with systemic sclerosis and smooth muscle actin positive (SMA +) HDFs^30^. In fibrotic mouse models, phosphorylation of c-Jun is associated with profibrotic cellular responses via activation of AKT^30^. However, we previously found that increased AKT phosphorylation by RL in HDFs inhibited migration and was associated with decreased collagen deposition^25,28^.

RL and near-infrared radiation stimulate cytochrome C oxidase in the mitochondria, altering mitochondrial membrane potential and increasing intracellular ATP and free-radical ROS^20,22^. We confirmed that ROS increases following irradiation with 320 and 640 J/cm^2^ RL using flow cytometry (Fig. 3E). ROS may alter the activity of fibrotic pathways, including TGF-β, mTOR, and AKT^22,25^. GO analysis demonstrated the enrichment of cellular responses to oxygen-containing compounds (GO: 1901701, p-value: 6.53 × 10^–5^, q-value: 1.67 × 10^–2^).

Transcription factor co-regulatory network analysis indicated that *RELA*, which codes for the p65 subunit of NF-κB, is predicted to regulate gene expression in RL-treated HDFs (Fig. 2G)^27,54^. The heterodimer of RELA and p50 is the most abundant form of NF- κB^27,54^. NF-κB is involved with inflammation and cellular responses to stress, including ROS^27,54^. NF-κB has been previously implicated in photobiomodulatory mechanism since low fluences of near-infrared light-activated NF-κB in mouse embryonic fibroblasts^55^.

Our primary objective was the discovery of RL induced transcriptome modulation. Two previous studies by Kim et al. and Li et al. examined the effects of RL on HDF transcription using RNA-Seq and demonstrated similar regulation of genes/pathways related to MMPs, FOS, JUN, NF-kB, SMAD1/7, oxidative stress, and inflammation^37,56^. Our comprehensive data set is a strength of this study and may serve as a reference for future research. This objective was limited by sparse prior research on photobiomodulation transcriptomics. This limitation restricts our enrichment analysis because photobiomodulation pathways may be under-represented in the major databases such as KEGG and GO. Our and others’ research may contribute to the identification of photobiomodulation pathways^37,56^. Another limitation of our study was that we did not use HDFs isolated from fibrotic tissue. However, fibrotic HDFs may lose their fibrotic phenotype after being removed from their in vivo fibrotic niche. Non-fibrotic HDFs may be a good analog as RL has similar anti-fibrotic effects in normal and keloid-derived HDFs^23,24^. In future research, the transcriptomic effects on fibrotic skin from reconstructed three-dimensional, animal, and clinical models should be assessed^57,58^. In vivo and tissue culture models may respond to RL phototherapy differently.

In conclusion, we identified several genes that may contribute to the mechanism of RL treatment of fibrosis. *MMP1* is a critical mediator of fibrotic disease that was modulated by RL treatment. We identified *PRSS35* as a potential mechanism of RL anti-fibrosis due to its 30-fold increased expression. *PRSS35* could be the focus of future photobiomodulation studies because of its previously limited characterization and profoundly differential expression in RL treated samples. Our results suggest that RL has the potential to benefit patients with fibrosis by altering gene expression.

## Methods

### Cell culture

Normal HDFs were obtained from the American Type Culture Collection (CRL-2617, CRL-2697, and CRL-2796) and Coriell Biorepository (AG13145). HDF Donor samples were used per relevant guidelines and regulations. HDFs were sub-cultured in DMEM (Invitrogen; Carlsbad, CA) supplemented with 10% FBS (R&D Systems; Minneapolis, MN) and 1% antibiotic/antimycotic (Invitrogen). Cells were maintained in a humidified incubator with 5% CO_2_ and 20% O_2_. RNA was collected from 35 mm tissue culture dishes (Corning, Corning, NY) that were initially seeded at low confluency (2 × 10^4^ cells total; 4,000 cells per 1.77 cm^2^ surface area) between passages four and seven^59^. Twenty-four hours after seeding, samples were treated with RL, and RNA was collected at 0, 4, 12, and 24 h time points.

### HDF donors

RNA-seq was performed with total RNA samples collected from four commercially available HDF cultures obtained from three different anatomical sites: two from the abdomen, one from the forearm, and one from the lower leg (Table 1).

### RL treatment

HDFs were treated with RL as previously described^23–25^. Briefly, an LED unit (Omnilux; Globalmed technologies, Napa, CA) was utilized for all experiments. The LEDs have a rectangular aperture with dimensions 4.7 cm × 6.1 cm and emit visible red light at 633 ± 30 nm wavelength in the electromagnetic spectrum. The light has a power density of 872.6 W/m^2^ at room temperature and 10 mm from the bottom of the plastic culture dish. Cell cultures were treated to 320 J/cm^2^ or 640 J/cm^2^ (3667 s for 320 J/cm^2^ and 7334 s for 640 J/cm^2^) of RL at approximately 34 ˚C. During RL treatments, the cells were exposed to environmental 20% O_2_ and 412 parts per million CO_2_ concentrations outside of the incubator. Controls were placed on plate warmers set to 34 ˚C and protected from light with aluminum foil to match RL treated samples' environmental conditions.

### RNA isolation

Total RNA from HDFs was collected at 0, 4, 12, and 24 h after RL treatment. The miRNeasy (Qiagen; Germantown, MD) kit was used to isolate RNA from cell cultures following the manufacturer’s suggested protocol. To briefly summarize, Qiazol reagent (Qiagen) was used to lyse cells, followed by chloroform extraction. The aqueous layer was obtained and mixed with 100% ethanol (Sigma; St. Louis). Spin columns further aided the separation of RNA and impurities from samples. Samples were treated with RNase-free DNase (Qiagen) to ensure no genomic DNA contamination. Finally, RNase free water was used to elute sample RNA. All samples had RNA quality assessed by Tapestation 2200 (Agilent Technologies; Santa Clara, CA). All samples had RNA integrity number values of 9.9 or 10.0.

### Library preparation and sequencing

RNA integrity was measured using the RNA Nano 6000 Assay Kit with the Agilent Bioanalyzer 2100 system (Agilent Technologies). Libraries were built with NEBNext Ultra Directional RNA Library Prep Kit for Illumina (New England Biolabs; Ipswich, MA). Sequencing of all samples was performed by pooling all of our indexed samples and putting equal amounts of the pooled sample into each lane of a flow cell performed on Hi Seq X (Illumina; San Diego, CA).

### Mapping and identification of differentially expressed genes

Sequencing reads were mapped to the UCSC human reference genome (GRCh37/hg19), and the following read counts were evaluated by STAR (version 2.5.2)^60^. Gene expression level normalization and differential expression analysis were carried out by DESeq2 (version 1.6.3) bioconductor R package^26^. To compare samples before and after treatment for different cell lines, a multifactor design was used applying DESeq2 controlling for the effect of cell line difference. Differential expression p-values were corrected for multiple testing using the false discovery rate (FDR) method. Enrichment analysis was performed with Enrichr^61,62^.

### RT-qPCR

RT-qPCR experiments used materials and equipment from Bio-Rad (Hercules, CA). 100 ng of RNA was synthesized into cDNA with the iScript reverse transcription kit using a C1000 thermal cycler. RT-qPCR was performed with 1 ng of cDNA on the BioRad CFX96 using SYBR green.

### ELISA

At 4, 12, and 24 h following RL irradiation, we quantified total human MMP-1 in collected HDF culture media using ELISA (R&D Systems) according to the manufacturer's guidelines. For each sample, the concentration of released MMP-1 was indexed to the total intracellular protein. We quantified collected intracellular protein using Bradford reagent (Bio-Rad). Optical density was measured for ELISA and protein concentration using a 96-well plate reader (Synergy 2, Biotek; Winooski, VT). For each donor, the experiment was performed in technical triplicate. Relative MMP-1 expression (RL/control) was pooled from the 4 donor lines and compared to a hypothetical mean of 1, indicating no difference between RL and control, using a one sample T-Test. P < 0.05 was considered significant. ** indicated p < 0.01.

### Cell count

Cell counts were assessed using crystal violet^63^. Following treatment with RL, experimental and control samples were placed in a humidified incubator for 48 h. Cells were fixed with 4% formaldehyde (Sigma) and stained with 0.1% crystal violet (Thermo-fisher Scientific; Waltham, MA). 10% acetic acid (Thermo-fisher Scientific) was used to elute the crystal violet. Optical density of eluted crystal violet was quantified with a plate reader at 595-nm. For each donor, the experiment was performed with a technical repeat of n = 3–5. Relative counts (RL/control) were pooled from the 4 donor lines and compared to a hypothetical mean of 1 (indicating no difference between RL and control), using a one sample T-Test. P < 0.05 was considered significant (*).

### Free radical reactive oxygen species generation

For free radical ROS generation, HDFs were assayed using dihydrorhodamine-123 (DHR-123; Thermo-fisher Scientific). Cells were irradiated with RL and then treated with DHR-123 for 30 min. Non-fluorescent DHR-123 converts to fluorescent rhodamine-123 in the presence of ROS. RL treated and control cells were detached with 0.25% trypsin EDTA (Thermo-fisher Scientific), collected, and analyzed with flow cytometry (Fortessa; BD; San Jose, CA). Intracellular ROS generation was assessed immediately following irradiation (0 hours). Positive control cells were treated with 0.6 mM hydrogen peroxide (Thermo-fisher Scientific) for 30 min. ROS was quantified as the median fluorescent intensity (MFI) of rhodamine-123 using FlowJo Software (BD). For each donor, the experiment was performed with a technical repeat of n = 4 or 5. Relative MFIs of rhodamine-123 (RL/control) were pooled from the 4 donor lines and compared to a hypothetical mean of 1 (indicating no difference between RL and control), using a one sample T-Test. P < 0.05 was considered significant (*).

## Supplementary Information

Supplementary Information 1.

Supplementary Information 2.

## Data Availability

The datasets generated during and/or analysed during the current study are included within manuscript or available from the corresponding author on reasonable request.
