# Supplementary material for: Transcriptome analysis of human dermal fibroblasts following red light phototherapy
Source: Sci Rep. 2021 Apr 1;11:7315. doi: 10.1038/s41598-021-86623-2 (PMC8017006; doi:10.1038/s41598-021-86623-2)

**Title:** Transcriptome analysis of human dermal fibroblasts following red light phototherapy

**Authors**: Evan Austin^1,2#^, Eugene Koo^1#^, Alexander Merleev^1^, Denis Torre^3^, Alina Marusina^1^, Guillaume Luxardi^1^, Andrew Mamalis^2^, Roslyn Rivkah Isseroff^1,4^, Avi Ma’ayan^3^, Emanual Maverakis^1^, Jared Jagdeo^1,2,4^*

**Affiliations:**

1. Department of Dermatology, University of California at Davis, Sacramento, CA, USA
2. Department of Dermatology, SUNY Downstate, Brooklyn, NY, USA
3. Department of Pharmacological Sciences, Mount Sinai Center for Bioinformatics, Icahn School of Medicine at Mount Sinai Health, New York, NY
4. Dermatology Service, Sacramento VA Medical Center, Mather, CA, US

**# Contributed equally to this work**

*** Corresponding author:**

Email: [jrjagdeo@gmail.com](mailto:jrjagdeo@gmail.com)

Figure S1. Upregulated GO pathway analysis for all timepoints, 640 J/cm^2^, only. Pathways are sorted by combined score. All pathways included were significantly upregulated with an adjusted q-value >.05.

Figure S2. Down-regulated GO pathway analysis for all timepoints, 640 J/cm^2^ only. Pathways are sorted by combined score. None of the pathways included were significantly downregulated with an adjusted q-value >.05.

Figure S3. Upregulated KEGG pathway analysis for all timepoints, 640 J/cm^2^, only. Pathways are sorted by combined score. Significantly upregulated pathways (adjusted q-value >.05) are above dashed-line.

Figure S4. Downregulated KEGG pathway analysis for all timepoints, 640 J/cm^2^, only. Pathways are sorted by combined score. Significantly Down-regulated pathways (adjusted q-value >.05) are indicated by a *.

Figure S5. DEGs associated with Fibrosis. Solid and dash lines indicate 640 and 320 J/cm^2^ conditions, respectively. Red lines are RL treated and blue lines are control.


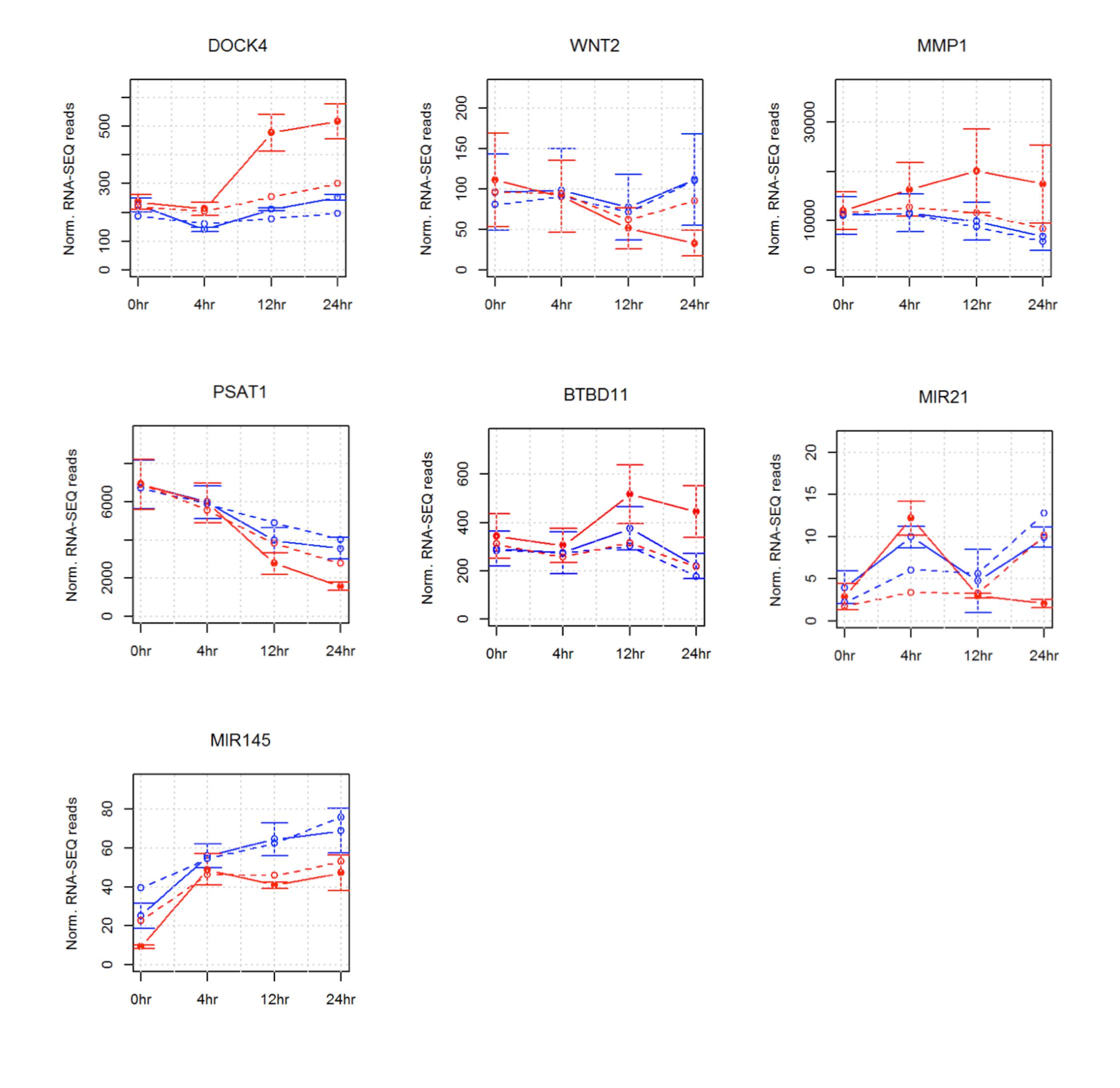


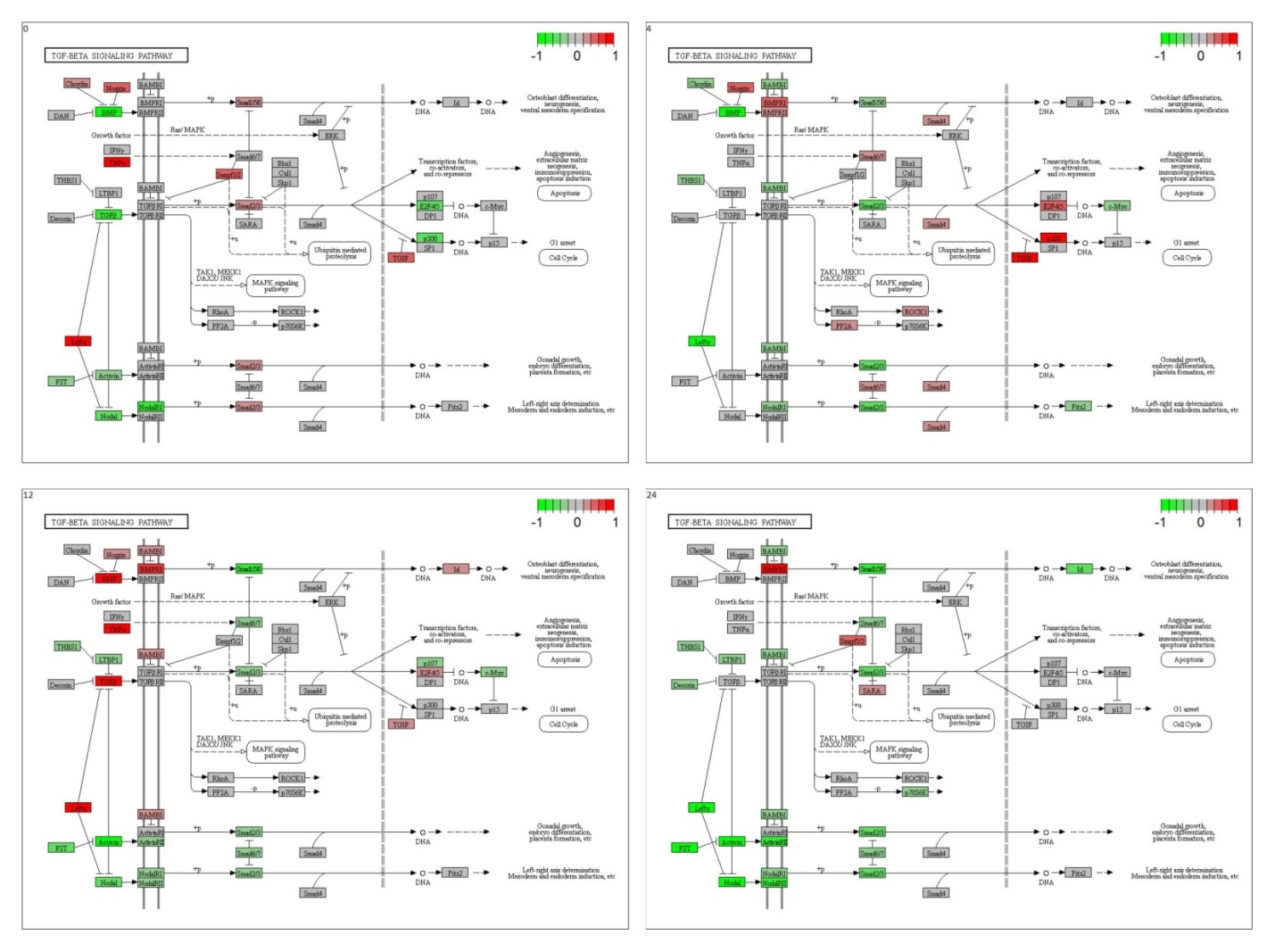


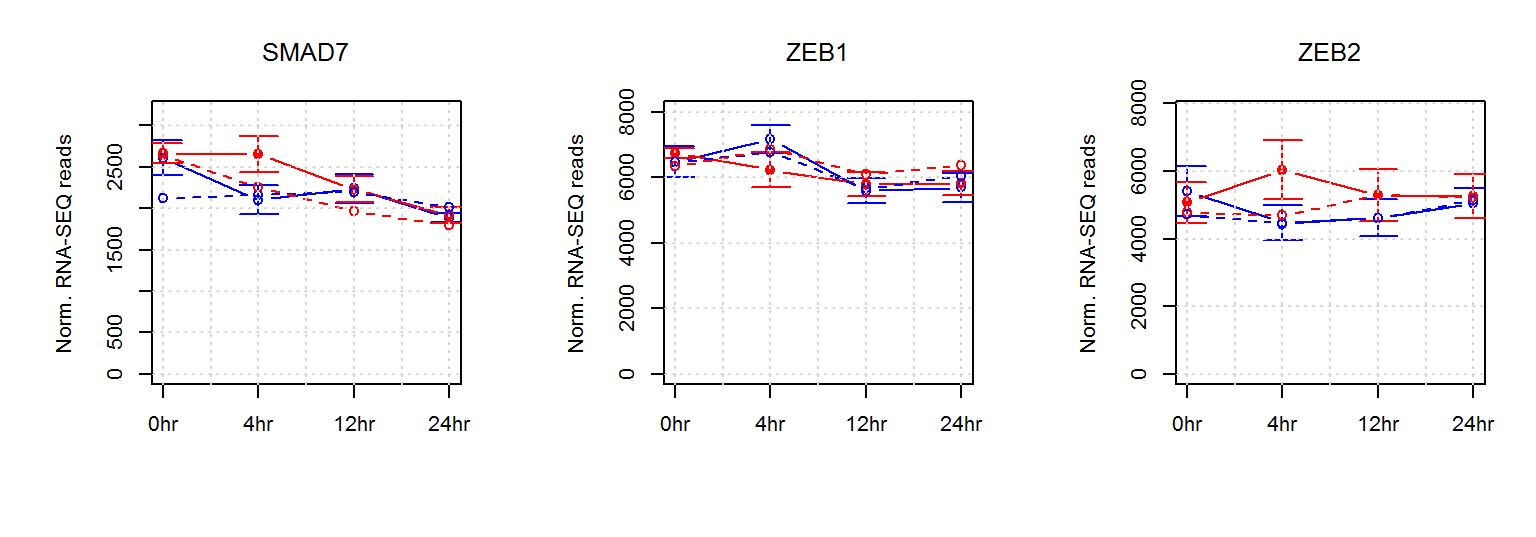

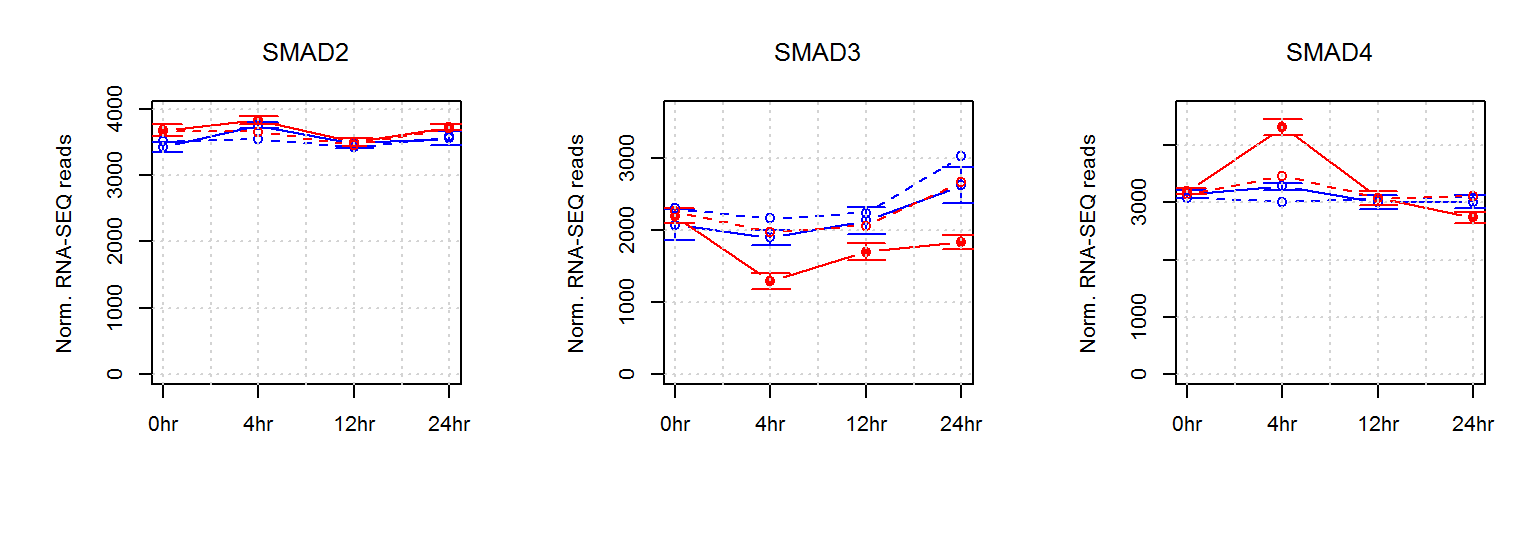


Figure S6. Differential expression of SMAD genes following RL phototherapy (A) TGF-β/SMAD signaling pathway map with respect to RNA-seq data. Genes in green are downregulated and genes in red are upregulated following RL irradiation. (B) SMAD DEGs. Solid and dash lines indicate 640 and 320 J/cm^2^ conditions, respectively. Red lines are RL treated and blue lines are control.

Figure S7. DEGs associated with ROS. Solid and dash lines indicate 640 and 320 J/cm^2^ conditions, respectively. Red lines are RL treated and blue lines are control.


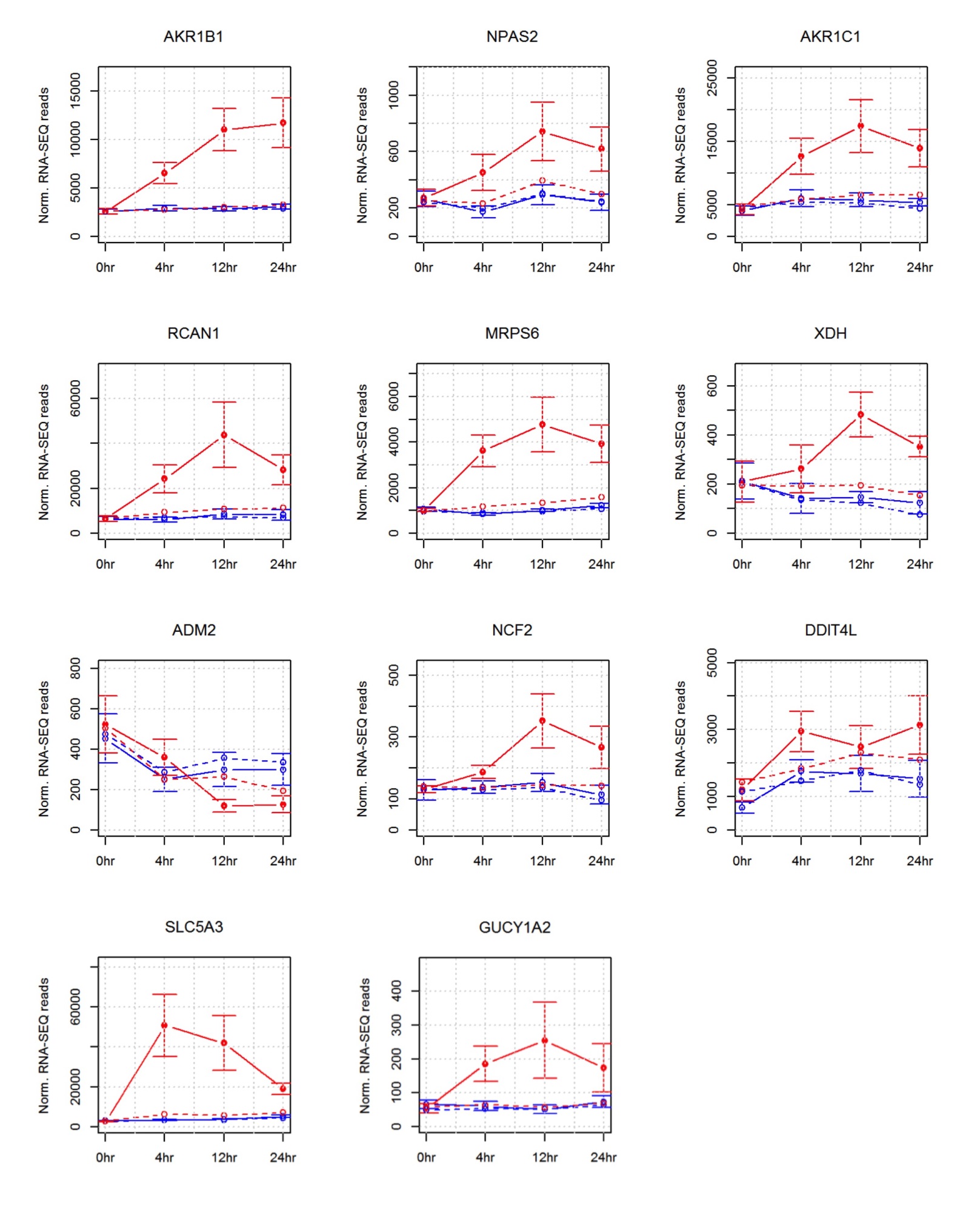

Supplement: Supplementary file 1 — Supplementary Information 1. [file 41598_2021_86623_MOESM1_ESM.docx]
